# Modulation of Cell Surface Protein Free Thiols: A Potential Novel Mechanism of Action of the Sesquiterpene Lactone Parthenolide

**DOI:** 10.1371/journal.pone.0008115

**Published:** 2009-12-02

**Authors:** Jolanta Skalska, Paul S. Brookes, Sergiy M. Nadtochiy, Shannon P. Hilchey, Craig T. Jordan, Monica L. Guzman, Sanjay B. Maggirwar, Margaret M. Briehl, Steven H. Bernstein

**Affiliations:** 1 James P. Wilmot Cancer Center, University of Rochester Medical Center, Rochester, New York, United States of America; 2 Department of Anesthesiology, University of Rochester Medical Center, Rochester, New York, United States of America; 3 Department of Microbiology and Immunology, University of Rochester Medical Center, Rochester, New York, United States of America; 4 Department of Pathology, Arizona Cancer Center, University of Arizona, Tucson, Arizona, United States of America; Bauer Research Foundation, United States of America

## Abstract

**Background:**

There has been much interest in targeting intracellular redox pathways as a therapeutic approach for cancer. Given recent data to suggest that the redox status of extracellular protein thiol groups (i.e. exofacial thiols) effects cell behavior, we hypothesized that redox active anti-cancer agents would modulate exofacial protein thiols.

**Methodology/Principal Findings:**

To test this hypothesis, we used the sesquiterpene lactone parthenolide, a known anti-cancer agent. Using flow cytometry, and western blotting to label free thiols with Alexa Fluor 633 C_5_ maleimide dye and N-(biotinoyl)-N-(iodoacetyl) ethylendiamine (BIAM), respectively, we show that parthenolide decreases the level of free exofacial thiols on Granta mantle lymphoma cells. In addition, we used immuno-precipitation techniques to identify the central redox regulator thioredoxin, as one of the surface protein thiol targets modified by parthenolide. To examine the functional role of parthenolide induced surface protein thiol modification, we pretreated Granta cells with cell impermeable glutathione (GSH), prior to exposure to parthenolide, and showed that GSH pretreatment; (a) inhibited the interaction of parthenolide with exofacial thiols; (b) inhibited parthenolide mediated activation of JNK and inhibition of NFκB, two well established mechanisms of parthenolide activity and; (c) blocked the cytotoxic activity of parthenolide. That GSH had no effect on the parthenolide induced generation of intracellular reactive oxygen species supports the fact that GSH had no effect on intracellular redox. Together these data support the likelihood that GSH inhibits the effect of parthenolide on JNK, NFκB and cell death through its direct inhibition of parthenolide's modulation of exofacial thiols.

**Conclusions/Significance:**

Based on these data, we postulate that one component of parthenolide's anti-lymphoma activity derives from its ability to modify the redox state of critical exofacial thiols. Further, we propose that cancer cell exofacial thiols may be important and novel targets for therapy.

## Introduction

There has been much recent interest in targeting intracellular redox signaling pathways as a therapeutic approach for cancer [1]–[3]. An integral component of these pathways is the thiol-disulfide oxidoreduction system whereby the function of proteins which contain free thiol groups (-SH) in their active or regulatory sites (i.e. transcription factors, molecular adapters, chaperones, protein tyrosine phosphatases and proteases) are regulated, in part, by thiol-disulfide oxidoreduction, often mediated through the thioredoxin-thioredoxin reductase and glutaredoxin-glutathione systems [4]. Finally, the activity of several anti-cancer agents, such as suberoylanilide hydroxamic acid (SAHA) and curcumin, are thought to work in part by modulating this pathway, inhibiting thioredoxin and thioredoxin reductase, respectively [5], [6].

Whereas there is much data to support the critical role that thiol-disulfide oxidoreduction plays in the regulation of intracellular processes, emerging data also suggests that cell surface protein thiols (i.e. exofacial thiols) are targets of redox regulation and that the redox status of such thiols regulates critical cellular functions. For example, the redox status of exofacial thiols on T-cells regulates their activation and proliferation [7]. Indeed, the redox status of critical thiols on the immunoglobulin superfamily member CD4, regulates both T-cell binding to antigen presenting cells as well as HIV-1 entry into CD4^+^ T-cells [8]. In fact, the levels of free exofacial thiols on normal donor lymphocytes differ from that seen from patients with HIV [9], [10]. In addition, the thiol redox status of integrin α-4 affects integrin-mediated cell adhesion [11]. Similarly, the thiol redox status of the tumor necrosis factor receptor superfamily member 8 (TNFRSF8/CD30) influences whether this receptor can engage its cognate ligand and transduce its downstream signaling in lymphocytes [12]. Finally various membrane receptors, ion channels [13] and extracellular thiol-disulfide oxidoreductases themselves are redox sensitive [14]. We therefore hypothesized that anti-cancer therapies may modulate the redox state of cancer cell exofacial thiols.

Parthenolide, a plant derived sesquiterpene lactone, has shown significant antitumor effects against human acute myeloid leukemia (AML) [15], acute and chronic lymphocytic leukemia (CLL) [16], and such solid tumors as breast [17], cholangiocarcinoma [18] and pancreatic cancer [19]. As a sesquiterpene lactone, it contains unsaturated double bonds conjugated with a carbonyl group (O = C–C = CH_2_) having nucleophilic properties which would be anticipated to react with free protein thiols *via* a Michael-like addition [20]. Therefore, we determined whether parthenolide modulates the redox state of lymphoma cell exofacial thiols.

## Materials and Methods

### Cell Culture and Chemical Reagents

All chemical reagents were purchased from Sigma (St. Louis, MO) unless otherwise stated. The human diffuse large B-cell lymphoma lines, SUD-HL6 [21] and OCI-LY19 [22], and the mantle cell lymphoma lines Granta [23], HF4B [24] and Rec-1 [25] were cultured as described [26]. The DLCL27B primary line (obtained from patient biopsy under a University of Rochester Institutional Review Board – approved protocol, was kindly provided by Randall Rossi, University of Rochester; informed consent was obtained in accordance with Declaration of Helsinki) was cultured in IMDM supplemented with 10% human plasma. All cells were maintained and treated at 37° and 5% CO_2_ unless otherwise noted. All cells were determined to be mycoplasma free using the MycoAlert® Mycoplasm Detection Kit (Lonza, Rockland, ME).

### Cell Viability Assay

Cells were treated with varying concentrations of parthenolide for 24 h and an 3-(4,5-dimethylthiazol-2-yl)-2,5-diphenyltetrazolium bromide (MTT) assay was then performed as described [26]. The results are presented as the percentage of cells that reduce MTT, as compared to DMSO-treated control cells. The interaction between parthenolide and MTT was tested, and no change in the absorbance of MTT was observed as a result of the addition of parthenolide in a cell free system (data not shown).

### Measurements of Reactive Oxygen Species Generation

Granta cells (10^6^/ml) were treated with antioxidant or vehicle for 1.5 h, stained with H_2_DCFDA (10 µM) (Invitrogen, Carlsbad, CA), treated with parthenolide (30 µM) or vehicle for an additional 15 to 30 min and then acquired on a 5-color upgraded FACScan cytometer (Becton-Dickinson, San Diego, CA and Cytek Development, Fremont, CA). H_2_DCFDA staining intensity was determined using FlowJo software version 7.2.4 (Tree Star, Ashland, OR).

### Cell Surface Free Thiol Measurements

Granta cells (10^6^/ml) were treated with antioxidant or vehicle for 2 h then exposed to parthenolide (30 µM) for an additional 3 h. Free surface thiol groups were determined using the Alexa Fluor 633 C_5_ maleimide dye (Invitrogen, Carlsbad, CA) as described [9]. Cells were acquired on a FACScan cytometer and analyzed using FlowJo software as above.

### Determination of Plasma Membrane Associated Thioredoxin

Granta cells were surface stained with a rabbit anti-thioredoxin primary antibody (dilution 1∶200 in PBS containing 1% FBS (PBS/FBS); Abcam, Cambridge, MA) followed by an Alexa Fluor 633 conjugated anti-rabbit secondary antibody (dilution 1∶200 in PBS/FBS; Invitrogen, Carlsbad, CA). Stained cells were harvested and acquired on a FACScan cytometer and analyzed using FlowJo software.

### Determination of Cell Surface Protein Thiols with BIAM

Granta cells (10^6^/ml) were treated with antioxidants or vehicle for 2 h followed by treatment with parthenolide (10 or 30 µM) or H_2_O_2_ (100 µM) for 3 h, followed by treatment with 100 µM BIAM (Invitrogen, Carlsbad, CA) as described [11]. A plasma membrane enriched fraction, obtained as described [9], was resolved by 15% SDS-PAGE (minimum of 20 µg of protein) transferred to a nitrocellulose membrane (BioRad, Hercules, CA) and probed with streptavidin-peroxidase (dilution 1∶2000; Sigma, St. Louis, MO) or specific antibodies. Purity of the plasma membrane enriched fractions was determined by western blot analysis using an anti-transferrin antibody (Zymed Lab. Invitrogen, Carlsbad, CA).

### PEGylation of Thioredoxin

Human, recombinant, N-terminal histidine tagged thioredoxin (Trx; ≈14kDa; 50 µg) was incubated with parthenolide (at the concentrations indicated in the legend to figures) for 30 min followed by incubation with MM(PEG)_24_ (150 µM; Pierce Biotech., Rockford, IL) for 30 min. The samples were then resolved by 17% SDS-PAGE, transferred to a nitrocellulose membrane (BioRad, Hercules, CA) and probed with rabbit anti-thioredoxin primary antibody (dilution 1∶200; Abcam, Cambridge, MA).

### Immunoprecipitation

Plasma membrane enriched cellular fractions from Granta cells (see Determination of Cell Surface Protein Thiols with BIAM section), were incubated in RIPA buffer, supplemented with 1 mM PMSF and Na_3_VO_4_, for 15 min followed by centrifugation. Supernatants were incubated with a 50% slurry of neutravidin-agarose (Pierce Biotech., Rockford, IL) as described [27] and the samples were resolved by 15% SDS-PAGE, transferred to a nitrocellulose membrane (BioRad, Hercules, CA) and probed against thioredoxin as above. To determine relative protein amount, a parallel electrophoresis was prepared, where the gel was stained with Coomassie Blue (data not shown).

### NFκB and JNK Determination

For detection of NFκB, JNK, and IκB-α (both phosphorylated and total), Granta cells were treated with GSH (5 mM) or vehicle for 2 h followed by treatment with parthenolide (30 µM) for 3 h. Treated cells were lysed with a buffer containing 10 mM HEPES, 1.5 mM MgCl_2_, 10 mM DTT, 0.5 mM PMSF and 0.1% NP-40 supplemented with a protease inhibitor cocktail (pH 7.9; Calbiochem, San Diego, CA). Proteins (20 µg) were resolved by 15% or 8% SDS-PAGE, transferred to a nitrocellulose membrane (BioRad, Hercules, CA) and probed with anti-phospho-NFκB p65 (dilution 1∶100; Cell Signaling, Danvers, MA), anti-NFκB p65 (dilution 1∶100; Cell Signaling, Danvers, MA), or anti-phospho-SPK/JNK (dilution 1∶100; Cell Signaling, Danvers, MA) rabbit polyclonal antibodies, anti-phospho-IκB-α (dilution 1∶100; Abcam, Cambridge, MA) or anti-IκB-α (dilution 1∶100; Santa Cruz Biotech, Santa Cruz, CA) rabbit polyclonal antibodies or, as a loading control, a mouse anti-actin antibody (dilution 1∶5,000; Calbiochem, San Diego, CA). Blots were detected with goat anti-rabbit or anti-mouse polyclonal antibodies conjugated with horseradish peroxidase (dilution 1∶10,000; Santa Cruz Biotech, Santa Cruz, CA).

### The Quantification of NFκB Activation

For determination of NFκB activation, Granta cells (3×10^7^) were treated with GSH (5 mM) or vehicle for 2 h followed by treatment with parthenolide (30 µM) for 3 h. Nuclear extracts from the cells were prepared and p65 DNA binding activity was measured using the TransAM™ NFκB kit according to the manufacturer's instructions (Active Motif, Carlsbad, CA).

### Statistical Analysis

The unpaired Student's *t*-test was used to determine the statistical difference between experimental and control groups. *P* values <0.05 were considered significant.

## Results

### Parthenolide Treatment Results in a Dose-Dependent Decrease in Lymphoma Cell Viability

**Figure 1:**
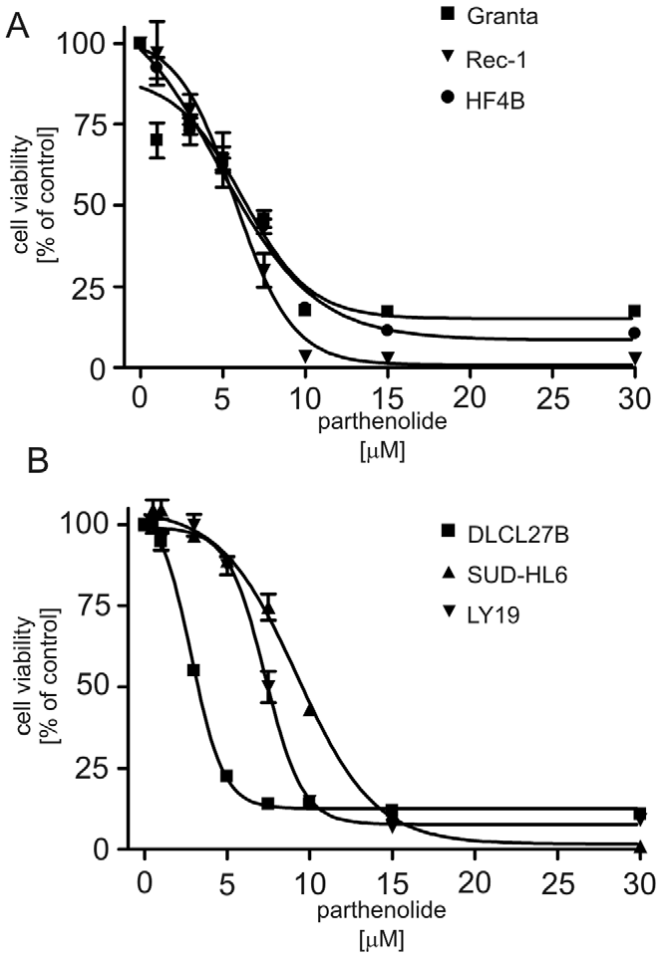
Parthenolide treatment results in a dose-dependent decrease in lymphoma cell viability. (*A*) Granta, Rec-1 and HF4B mantle cell lymphoma cells or (*B*) DLCL 27B, SUD-HL6 and OCI-LY19 diffuse large cell lymphoma cells were treated with the indicated concentrations of parthenolide for 24 h and cell viability measured by MTT assay. The results are expressed as mean ± s.d. (*n* = 6).

To determine the effect of parthenolide on mantle cell lymphoma (MCL) and diffuse large B-cell lymphoma (DLCL) viability, MTT assays were performed after 24 h exposure of MCL and DLCL cells to increasing doses of parthenolide. As shown in Fig. 1*A*, the viability of Granta, Rec-1 and HF4B cells (MCL cell lines) decreased in a dose-dependent fashion after exposure to parthenolide, with an EC_50_ of 7.5±0.2, 5.3±0.2 and 5.2±0.3 µM, respectively. Parthenolide similarly decreased the viability of the DLCL cell lines SUD-HL6 and OCI-Ly19, with an EC_50_ of 9.3±0.4 and 7.3±0.3 µM, respectively, (Fig. 1*B*). Parthenolide also had a potent dose-dependent effect on the viability of DLCL cells cultured directly from a patient biopsy specimen (DLCL 27B) having an EC_50_ = 3.0±0.11 µM (Fig. 1*B*). In our subsequent studies, we used the Granta MCL line to elucidate the mechanism of parthenolide action.

### Parthenolide Attenuates the Levels of Exofacial Free Thiols as Assessed by Flow Cytometry

**Figure 2:**
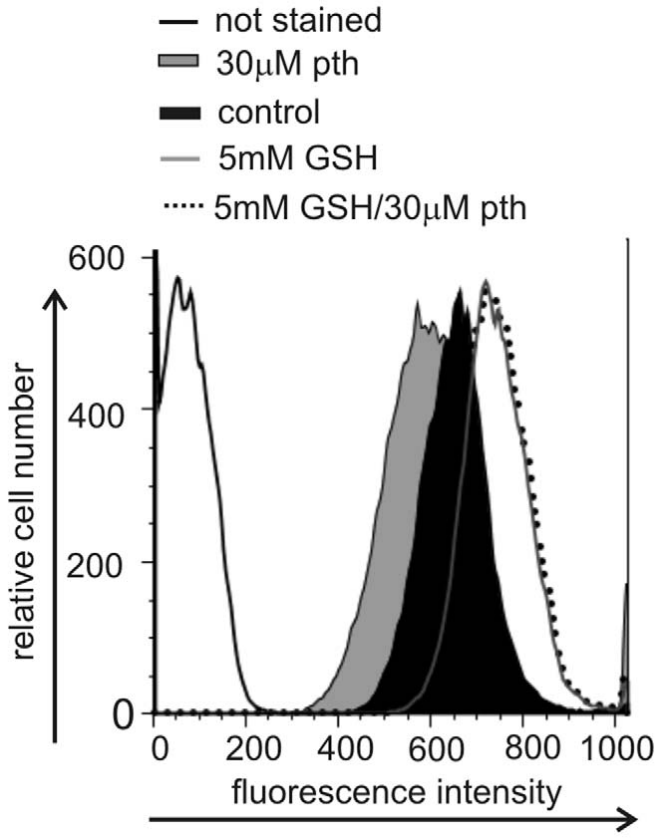
Parthenolide attenuates the levels of exofacial free thiols as assessed by flow cytometry. The change in Granta cell free exofacial thiol groups was assessed by staining cells with 1 µM Alexa 633 maleimide with analysis by flow cytometry after exposure to; 30 µM parthenolide alone for 3 h; 5 mM GSH alone for 2 h; 5 mM GSH for 2 h followed by 30 µM parthenolide for 3 h; or no treatment (control).

To directly assess the effect of parthenolide on Granta cell exofacial thiols we used an Alexa Fluor 633 coupled maleimide compound (ALM). Maleimide is a nucleophile that covalently binds to free thiol groups, and when coupled with the charged Alexa Fluor 633 dye becomes cell impermeable, allowing for the examination of cell surface thiol levels. As shown in Fig. 2, exposure of Granta cells to 30 µM parthenolide for 3 h resulted in a decrease in exofacial free thiols by approximately 36% relative to that of vehicle treated control cells. In contrast, treatment with cell-impermeable glutathione (GSH) increased the levels of exofacial free thiols by approximately 49% (Fig. 2). When cells were pretreated with GSH for 2 h, washed and exposed to 30 µM parthenolide for 3 h there was no parthenolide induced decrease in exofacial free thiol levels (Fig. 2). Pretreatment with the cell permeable monoethyl ester (GSHee) or the GSH precursor *N*-acetyl-L-cysteine (NAC) similarly protected exofacial thiols from modification by parthenolide (data not shown). The histogram shown in Fig. 2 is representative of 3 independent experiments. The MFI (mean fluorescent intensity) of the control cells from all 5 experiments varied only by +/− 8%, and the direction of each experimental result in relationship to that of the control cells was the same in each experiment. Taken together, the data suggest that parthenolide modulates exofacial free thiol groups; a process that is inhibited by pretreatment with GSH.

To control for the possibility that GSH pretreatment alters intracellular redox, which would result in an increase in surface free thiols, we evaluated the effect of GSH on parthenolide induced ROS generation (Supplementary Fig. S1). Whereas pretreatment for 2 h with 5 mM of cell permeable GSHee or NAC partially inhibited parthenolide induced ROS generation, a 2 h pretreatment with 5 mM GSH had no effect on intracellular ROS generation. Similar results were seen using parthenolide at doses of 5 and 10 µM for 15, 30 and 60 min of exposure (data not shown). If GSH was altering intracellular redox it should have attenuated parthenolide induced ROS generation similar to that seen with GSHee and NAC. Taken together, these data suggest that the effect of GSH on exofacial free thiols is a direct effect and not due to an effect on intracellular redox.

To control for the possibility that exofacial thiol modification is a result of intracellular H_2_O_2_, generated by parthenolide, which would be anticipated to diffuse across the cell membrane and directly modify exofacial thiols, we used the cell impermeable H_2_O_2_ scavenger, catalase. Exposure of Granta cells to 5 mM H_2_O_2_ resulted in exofacial protein oxidation, (as demonstrated by Alexa Fluor staining; Supplemental Fig. S2 *A*), which is blocked by 500 µM catalase. In contrast, exposure of Granta cells to 30 µM parthenolide results in exofacial protein oxidation, which is not blocked by catalase (Supplemental Fig. S2 *B*). Taken together, parthenolide is not modifying exofacial thiols through an H_2_O_2_ dependent mechanism.

### Parthenolide Attenuates the Levels of Exofacial Free Thiols as Assessed by Western Blot Analysis

**Figure 3:**
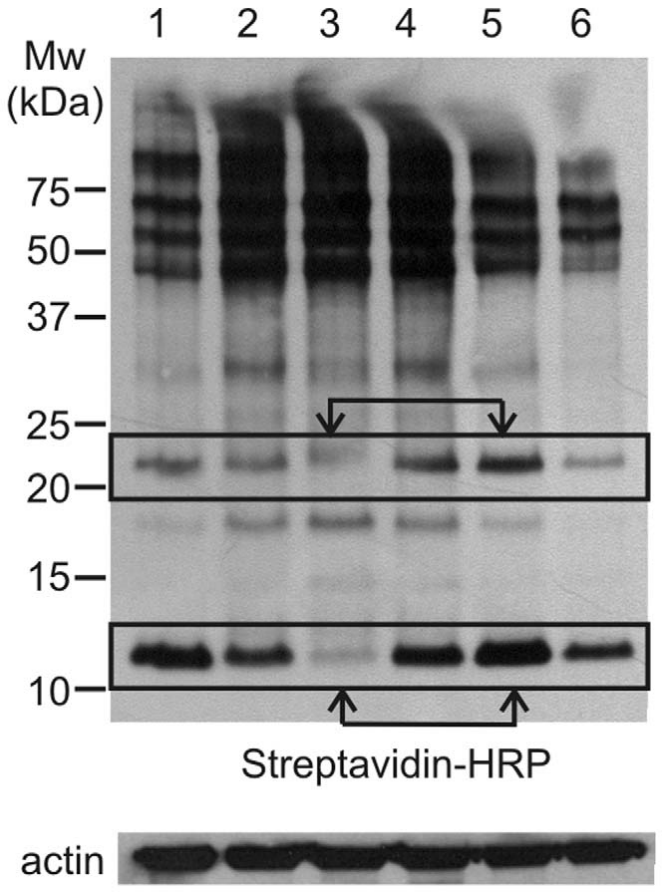
Parthenolide attenuates the levels of exofacial free thiols as assessed by western blot analysis. Plasma membrane enriched cell lysates were obtained from Granta cells treated as follows: (*lane 1*) untreated control cells; (*lane 2*) 10 µM parthenolide for 3 h; (*lane 3*) 30 µM parthenolide for 3 h; (*lane 4*) 5 mM GSH alone for 2 h; (*lane 5*) 5 mM GSH for 2 h followed by 30 µM parthenolide for 3 h; (*lane 6*) 100 µM H_2_O_2_ for 3 h. The lysates were stained with the thiol probe BIAM and blotted with streptavidin-HRP. As a protein loading control the blots were also probed for actin (bottom panel).

To further confirm that parthenolide modifies exofacial free thiols, we used a biotinylated thiol reactive reagent, N-(biotinoyl)-N-(iodoacetyl) ethylendiamine (BIAM), which binds to free thiol groups and is detected by streptavidin-peroxidase staining of western blots. As shown in Fig. 3, *lane 1*, plasma membrane enriched cell lysates from untreated Granta cells contain significant numbers of proteins having free thiol groups (with each band corresponding to a protein having a free thiol group able to interact with BIAM). After exposure of Granta cells to 10 (Fig. 3, *lane 2*) or 30 µM (Fig. 3, *lane 3*) parthenolide for 3 h, however, there is a dose dependent decrease in the intensity of the bands corresponding to ∼22kD and ∼12kD suggesting that parthenolide is modifying the free thiols of these proteins, making them unreactive to BIAM.

We next determined whether the cell impermeable GSH attenuated the effect of parthenolide on the free thiols of the ∼22 and∼12kD proteins. Treatment of Granta cells with 5 mM GSH alone (Fig. 3, *lane 4*) had no significant effect on the intensity of the bands corresponding to the ∼22 and ∼12kD proteins as compared to that seen in untreated control cells (Fig. 3, *lane 1*). In contrast, a 2 h pre-treatment with 5 mM GSH followed by exposure to 30 µM parthenolide for 3 h significantly attenuates the decrease in the intensity (and thus modification) of the bands corresponding to the ∼22 and ∼12kD proteins seen with a 3 h exposure of 30 µM parthenolide alone (compare Fig. 3 *lane* 3, parthenolide alone, with *lane* 5, GSH plus parthenolide).

As a control for the oxidation of protein free thiols, H_2_O_2_ was used. Exposure of Granta cells to H_2_O_2_ resulted in decreased signals in multiple bands that included the ∼22kD and ∼12kD bands (Fig. 3, *lane 6*) modified by parthenolide. Equal protein loading in each lane was demonstrated with actin staining. Membrane purity is shown by blotting with an anti-transferrin antibody (Supplementary Fig. S3). Taken together with the flow cytometric data presented in Fig. 2, parthenolide modulates exofacial free thiols, including those of proteins having molecular weights of ∼22kD and ∼12kD, and such modifications are blocked by pretreatment with GSH.

### Identification of Surface Thioredoxin-1 as One of the Targets of Parthenolide

**Figure 4:**
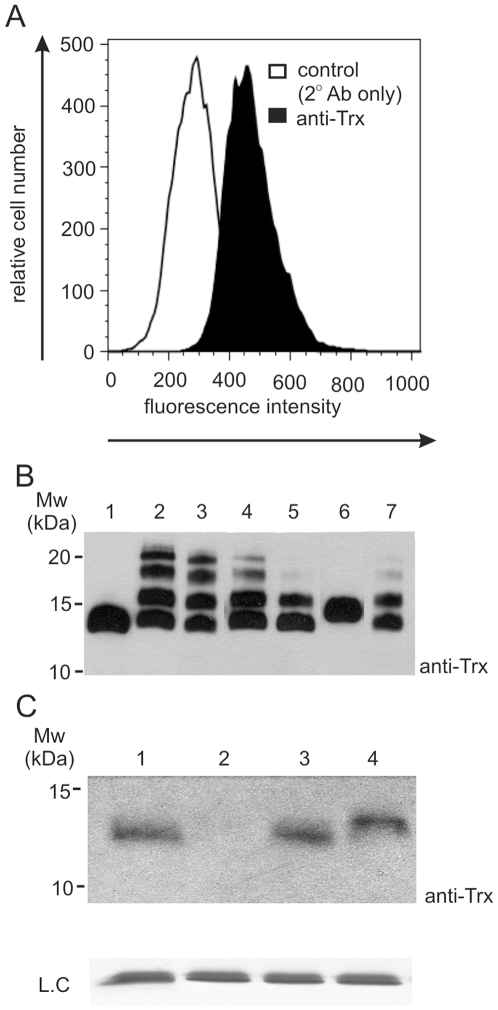
Identification of surface thioredoxin-1 as one of the targets for parthenolide. (*A*) Granta cells were surface stained for thioredoxin-1 and labeled with secondary antibody (anti-thioredoxin-1) or incubated with secondary antibody alone (control), and subjected to flow cytometric analysis. (*B*) Human purified thioredoxin-1 (14kD; *lane 1*) was co-incubated with MM(PEG)_24_, without (*lane 2*) or with pretreatment with 5 (*lane 3*), 10 (*lane* 4), 30 (*lane 5*) or 50 µM parthenolide (*lane 6*). As a control for free thiol group specific staining, thioredoxin-1 was pretreated with 10 µM of the thiol alkylating agent N-ethylmaleimide (NEM) prior to incubation with MM(PEG)_24_ (*lane 7*). The samples were then blotted and probed with a thioredoxin-1 antibody. (*C*) BIAM labeled proteins from plasma membrane enriched fractions were precipitated using neutravidin-agarose resins, which have a strong affinity for biotinylated proteins. The following conditions were tested on Granta cells; untreated (*lane 1*), treated with 30 µM parthenolide for 3 h (*lane 2*), 5 mM GSH for 2 h (*lane 3*), or with 5 mM GSH for 2 h and then 30 µM parthenolide for additional 3 h (*lane 4*). The cells were stained with BIAM and proteins having reduced thiol groups were precipitated with Neutravidin-agarose and probed with a thioredoxin-1 antibody. As a loading control (L.C.) the proteins on the gel were stained with Coomassie blue and shown is the ∼12kD band.

We hypothesized that one of the candidate proteins modified by parthenolide was thioredoxin-1, a 12kD protein containing thiol groups amenable to parthenolide modification. We first evaluated whether thioredoxin-1 can be detected on the Granta cell surface. Granta cells were stained with an anti-thioredoxin antibody and analyzed by flow cytometry (Fig. 4*A*). The mean fluorescent intensity (MFI) of staining with anti-thioredoxin antibody is approximately 85% greater than that of the secondary antibody alone, confirming that thioredoxin is on the Granta cell surface.

We next determined whether parthenolide could directly interact with free thiol groups within purified human thioredoxin-1. Thioredoxin-1 was co-incubated with maleimide conjugated to a polyethylene glycol spacer having terminal methyl groups (MM(PEG)_24_). Maleimide binds to free thiols on thioredoxin and upon SDS-PAGE separation and blotting with an anti-thioredoxin antibody gives a characteristic ladder pattern which is a mixture of thioredoxin with different molecular weights due to MM(PEG)_24_ conjugation to ≥1 of the 4 free thiols of thioredoxin (Fig. 4*B*, *lane 2*). However, when thioredoxin was pre-incubated with different concentrations of parthenolide (ranging from 5 to 50 µM), the sites available for MM(PEG)_24_ disappeared in a dose dependent manner (as evidenced by a decrease in laddering; Fig. 4*B*, *lanes 3−5*), and were completely abolished in the presence of 50 µM parthenolide (Fig. 4*B*, *lane 6*). Taken together, parthenolide directly binds to free thiol groups on thioredoxin-1. As expected, co-incubation of thioredoxin-1 with the thiol alkylator, N-ethylmaleimide (NEM), also reduced the sites available for MM(PEG)_24_ binding thus decreasing thioredoxin laddering (Fig. 4*B*, *lane 7*).

Finally, we determined whether parthenolide was directly modifying membrane associated thioredoxin-1 in Granta cells by BIAM staining and neutravidin pull-down. As shown in Fig. 4*C*, *lane 1*, one of the biotinylated proteins pulled down by neutravidin is thioredoxin. However, when Granta cells were treated with 30 µM parthenolide for 3 h, and then treated with BIAM, none of the proteins pulled down by neutravidin reacted with the anti-thioredoxin antibody (Fig. 4*C*, *lane 2*), suggesting that parthenolide has modified thioredoxin free thiols, making them unavailable to BIAM binding and thus they are not pulled-down by neutravidin. In contrast, when cells were first treated with 5 mM GSH for 2 h, and then exposed to 30 µM parthenolide for 3 h, the pulled down proteins were recognized by the anti-thioredoxin antibody (Fig. 4*C*, *lane 4*), suggesting that the free thiol groups were protected from parthenolide (via an as yet unknown mechanism), and thus available for interaction with BIAM. Lastly, GSH treatment alone had no effect on the thioredoxin pull-down (Fig. 4*C*, *lane 3*).

To exclude the possibility that the effect of parthenolide on thioredoxin free thiols was an indirect effect, due to a change in the pH of the buffer elicited by parthenolide (and the thiol modifications thus due to the change in pH), we evaluated the pH in the buffer, in the presence and absence of parthenolide, with both color pH indicator strips and a pH meter. Parthenolide had no effect on the pH of the buffer (data not shown).

### Parthenolide Cytotoxicity Is Blocked by Thiol Antioxidants

**Figure 5:**
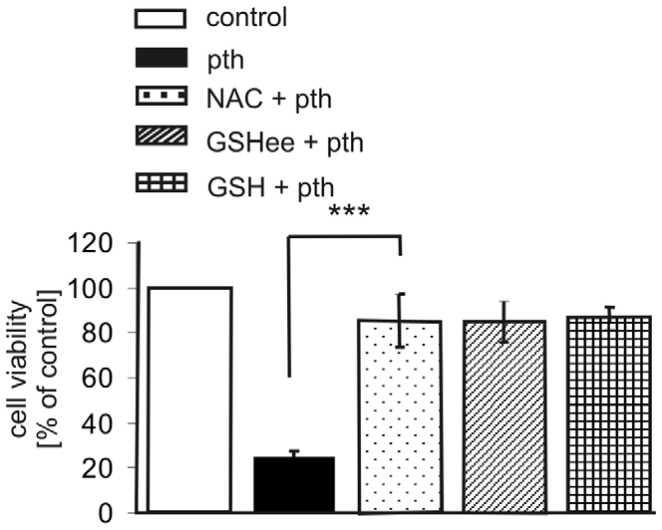
Parthenolide cytotoxicity is blocked by pre-treatment with thiol antioxidants. Where indicated, Granta cells were treated with parthenolide alone for 3 h or with NAC (5 mM), GSHee (5 mM) or GSH (5 mM) for 2 h, washed and then treated with 30 µM parthenolide for an additional 3 h, and cell viability determined by MTT 11 h later. *** p<0.05. The experimental points are the mean ± s.d. of six independent experiments.

To gain insight into whether the parthenolide induced reduction in free exofacial thiols is a mechanism through which parthenolide mediates its cytotoxic effect, we next determined whether the effect of parthenolide on Granta cell death can be blocked by pretreatment with GSH, GSHee and NAC using the same conditions that blocked the parthenolide induced decrease in exofacial free thiols. As parthenolide can form covalent adducts directly with GSH and NAC (data not shown), the antioxidants were extensively washed out prior to the addition of parthenolide. As shown in Fig. 5, a 3 h exposure of Granta cells to 30 µM parthenolide was sufficient to result in a significant decrease in cell viability after 14 h (25% of control as measured by MTT assay), although there was no significant cell death seen within the 3 h duration of treatment itself (data not shown). When cells were pretreated with thiol antioxidants for 2 h, the antioxidants washed out, and the cells exposed to 30 µM parthenolide for 3 h, cell viability increased from 25% without antioxidant pretreatment to approximately 85% with 5 mM NAC, 5 mM GSEee or 5 mM GSH pretreatment, respectively (Fig. 5). Similar results were seen when the cells were exposed to 10 µM parthenolide (data not shown). In contrast, when Granta cells were pretreated with parthenolide (10 or 30 µM) for 3 h followed by antioxidant exposure, the cytotoxic effects of parthenolide were not inhibited (data not shown).

### NFκB Antagonism and JNK Activation by Parthenolide Is Blocked by GSH

As parthenolide has been shown to induce malignant cell death by inhibiting NFκB activation and/or activating JNK [15], [17], we next determined whether GSH, which blocked the effect of parthenolide on exofacial thiols, could alter the effect of parthenolide on NFκB and JNK.

**Figure 6:**
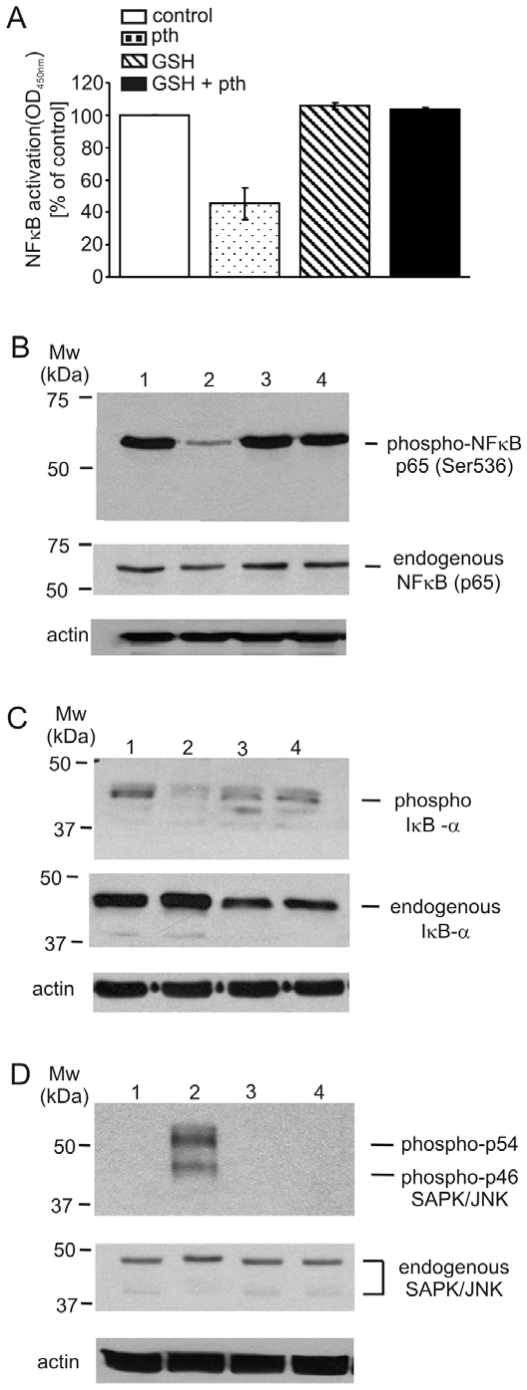
Parthenolide inhibition of NFκB and activation of JNK is inhibited by GSH. (*A*) NFκB activity is inhibited by parthenolide as assessed by TransAM NFκB kit (*Active Motif*). NFκB activation (measured as OD450 nm and presented as % of that of the non-treated control cells); of Granta cells treated with 30 µM parthenolide for 3 h; 5 mM GSH alone; 5 mM GSH for 2 h followed by parthenolide 30 µM for 3 h. (*B–D*) Western blot analysis of extracts from Granta cells; un-treated (*lane 1*), treated with 30 µM parthenolide for 3 h (*lane 2*), 5 mM GSH alone for 2 h (*lane 3*), and 5 mM GSH for 2 h, washed and treated with 30 µM parthenolide for additional 3 h (*lane 4*), using anti-phospho-NFκB p65 (Ser536) or anti-NFκB (p65) rabbit Abs (*B*, *upper* and *middle* panels, respectively); anti-phospho-IκB-α or anti-IκB-α rabbit Abs (*C*, *upper* and *middle* panels, respectively); an anti-phospho-SPK/JNK (Thr183/Tyr185) rabbit Ab or anti-SAPK/JNK mouse Ab (*D*, *upper* and *middle* panels, respectively). As a protein loading control, blots were probed for actin levels (*B–D*, *bottom* panels).

Using a highly sensitive and specific ELISA-based assay that detects only activated NFκB subunits [28], exposure of Granta cells to 30 µM parthenolide for 3 hours resulted in an approximately 5-fold decrease in p65 DNA binding activity (Fig. 6*A*). These inhibitory effects of parthenolide on p65 DNA binding activity, however, were reversed by preincubating the cells with 5 mM GSH (Fig. 6*A*).

Since the post-translational modification of p65 often determines the transcriptional activity of this molecule, we next evaluated the effect of parthenolide on the key phosphorylation site, serine 536, within the p65 molecule. Exposure of Granta cells to 30 µM parthenolide for 3 hours resulted in a significant decrease in the phosphorylation state of p65 (Fig. 6*B*, *lane 2*) which was abrogated by pretreatment with 5 mM GSH for 2 hours (Fig. 6*B*, *lane 4*). That the total p65 levels under these experimental conditions remained unaltered suggests that the findings shown in Fig. 6*B* are truly due to the modification of p65 by such experimental conditions.

As the activation of NFκB is intimately coupled with the signal induced phosphorylation and subsequent proteosomal degradation of the NFκB inhibitor, IκB-α, we next evaluated the effect of parthenolide on phospho- and total IκB-α levels. As shown in Fig. 6*C*, exposure of Granta cells to 30 µM parthenolide for 3 hours resulted in a decrease in phospho-IκB-α, which is highly consistent with the observed effects of parthenolide on p65 DNA binding activity and modification of p65 (Fig. 6*A, and 6B*, respectively). It is of interest that the levels of total endogenous IκB-α were decreased in the cells exposed to GSH (Fig. 6*C*, *lanes 3* and *4*). This may be a result of the reported effects of GSH on IκB-α degradation [29]

Finally the effect of GSH on parthenolide-mediated activation of JNK was examined by conducting immunoblot analyses. Analogous to the observations outlined in the above experiments, parthenolide-dependent phosphorylation of JNK isoforms (p46 and p54) was completely blocked in the cells pre-treated with GSH (Fig. 6*D*).

## Discussion

The studies herein demonstrate that exposure of human mantle and diffuse large cell lymphoma cells to parthenolide results in a decrease in free exofacial thiols of proteins having molecular weights of ∼12kD and ∼22kD. It is of interest that parthenolide does not have a global effect on all free exofacial thiols but rather a specific effect on exofacial thiols of these ∼12 and ∼22kD proteins. Whether this is due, for example, to differences in the protonation state of the different surface protein thiols and/or to structural constraints of the proteins that alter the access parthenolide has to the free thiols groups is not known.

We further identify the critical redox regulator thioredoxin-1 as one such protein. In addition, we show that pretreatment of Granta cells with cell impermeable GSH inhibits this parthenolide induced decrease in exofacial thiols. We further show that GSH inhibits parthenolide inhibition of NFκB and activation of JNK, as well as parthenolide induced cell death. We therefore postulate that one mechanism of the anti-lymphoma activity of parthenolide may be modulation of exofacial thiols.

Alternative explanations for the above findings are that; (a) despite extensive washout of GSH before exposure of Granta cells to parthenolide, residual GSH binds to and prevents intracellular accumulation of parthenolide and the subsequent elicitation of cell death and; (b) GSH may result in an increase in intracellular GSH, which itself would result in an increase in free exofacial thiols. If either of these were true, however, then GSH pre-treatment of Granta cells would be expected to inhibit parthenolide induced ROS generation, which it does not (Supplementary Fig. S1).

It is also possible that the increased free surface thiols generated by GSH pretreatment simply sequester parthenolide, preventing its intracellular accumulation. If this was the case however, these free thiol groups generated by GSH (which would bind the Alexa Fluor 633 C_5_ maleimide dye and thus fluoresce) would be modified by parthenolide (and would not bind the Alexa 633 maleimide and thus not fluoresce), and as such the MFI of the GSH followed by parthenolide treated cells would be less then that of the GSH alone treated cells. In contrast, the level of free exofacial thiols after exposure of cells to GSH followed by parthenolide is similar to that seen after exposure of the cells to GSH alone (Fig. 2). How GSH protects free exofacial thiols from modification by parthenolide is not clear, but it may disrupt surface protein intra- and/or inter-protein disulfide bonds, resulting in structural changes that make the free thiol targets of parthenolide inaccessible to the drug.

The mechanism whereby altering the redox state of exofacial thiols modulates intracellular events that result in cell death is not clear. It is possible that surface thioredoxin, which we show is one target of parthenolide, may mediate the cross-talk between exofacial thiols and downstream intracellular events. For example, thiol modification of surface thioredoxin results in its lipid raft-mediated internalization resulting in changes in the intracellular redox state [30]. Extracellular thioredoxin has also been shown to regulate receptor-ligand signaling interactions, specifically the binding of CD30 to its cognate ligand [12]. In addition, certain ion channels (i.e. Ca^2+^ channels) are activated as a result of the disruption of extracellular disulfide bridges by thioredoxin [14]. Taken together, the effect of parthenolide on surface thioredoxin may modulate intracellular pathways.

Parthenolide has been shown to have pre-clinical activity in AML [15], CLL [16], as well as multiple solid tumors [17]–[19]. The effect of parthenolide is relatively tumor selective, having no effect on normal hematopoietic stem cells or T-cells [15]. There has been recent evidence to suggest that in contrast to the oxidative extracellular redox state of normal cells, malignant cells exist in a reduced extracellular environment [31]. This raises the possibility that the tumor selectivity of parthenolide is due, in part, to this difference in extracellular redox state. For example, it is possible that the exofacial thiol targets of parthenolide are reduced on cancer cells, and thus are free to interact with the drug, but are oxidized on normal cells, thus unavailable for interaction with parthenolide.

In summary, we show that parthenolide modulates cancer cell exofacial thiols, including that of surface thioredoxin. That blocking parthenolide modulation of such thiols also blocks parthenolide induced cell death raises the possibility that one mechanism for the anticancer activity of parthenolide is through exofacial thiol modification. Indeed, the identification of such targets of parthenolide, and a determination of whether these targets differ in their redox status in normal compared to malignant cells, will not only provide further insights into cancer biology but will identify additional targets for development of specific anti-cancer therapies.

## Supporting Information

Figure S1 Generation of ROS in Granta cells as assessed by H2DCFDA. The level of ROS in Granta cells after 15 min exposure to 30 µM parthenolide was measured alone and after 2 h preincubation with 5 mM GSHee (A), 5 mM GSH (B) and 5 mM NAC (C). The results are representative of three independent experiments. (C).
(8.33 MB TIF)

Figure S2 The effect of parthenolide on exofacial free thiols is not a consequence of H2O2 oxidation. (A) The change in Granta cell free exofacial thiol groups was assessed by staining cells with 1 µM Alexa 633 malemide with analysis by flow cytometry after exposure to 5 mM H2O2; 500 µM catalase (cat) and 5 mM H2O2 for 3 h; and vehicle (control). (B) The change in Granta cell free exofacial thiol groups was assessed as described above after exposure to 30 µM parthenolide (pth) for 3 h; 500 µM catalase (cat) and 30 µM parthenolide for 3 h; and vehicle (control). The graphs are representative of 3 independent experiments.
(8.35 MB TIF)

Figure S3 Western blot analysis of plasma membrane fractions. Samples corresponding to plasma membrane enriched fractions (lane 1), and cytoplasmic fractions from Granta cells (lane 2), were resolved by SDS-PAGE (20 µg of proteins), transferred and probed against transferrin receptor (membrane protein) or actin.
(8.52 MB TIF)
